# Lyme carditis in Europe

**DOI:** 10.3389/fmicb.2026.1905414

**Published:** 2026-07-30

**Authors:** Dragana Gazibara, Maria Pete, Lidija Popović-Dragonjić, Tatjana Miljković, Milovan Petrović, Pavle Banović

**Affiliations:** 1 Medical Faculty of Novi Sad, University of Novi Sad, Novi Sad, Serbia; 2 Pasteur Institute Novi Sad, Novi Sad, Serbia; 3 Balkan Association for Vector-Borne Diseases, Novi Sad, Serbia; 4 Clinic for Infectious Diseases, University Clinical Center of Vojvodina, Novi Sad, Serbia; 5 Clinic for Infectology, University Clinical Center, Niš, Serbia; 6 Department of Infectious Diseases and Epidemiology, Faculty of Medicine, University of Niš, Niš, Serbia; 7 Institute of Cardiovascular Diseases of Vojvodina, Sremska Kamenica, Serbia; 8 Department of Prevention of Rabies and Other Infectious Diseases, Clinic for Lyme Borreliosis and Other Tick-Borne Diseases, Pasteur Institute Novi Sad, Novi Sad, Serbia; 9 Department of Microbiology with Parasitology and Immunology, Faculty of Medicine in Novi Sad, University of Novi Sad, Novi Sad, Serbia

**Keywords:** atrioventricular block, *Borrelia burgdorferi sensu lato*, Europe, *Ixodes ricinus*, Lyme borreliosis, Lyme carditis, pacemaker implantation, surveillance

## Abstract

Lyme borreliosis is the most common tick-borne bacterial infection in Europe and is caused by members of the *Borrelia burgdorferi sensu lato* complex transmitted mainly by *Ixodes ricinus*. Although Lyme carditis represents an uncommon extracutaneous manifestation, it is clinically important because atrioventricular conduction disease can progress rapidly yet often resolves with timely antibiotic treatment, reducing avoidable permanent pacemaker implantation. This Mini Review synthesizes the European evidence on the microbiological, epidemiological, diagnostic, and surveillance dimensions of Lyme carditis. The European setting is distinct from North America because *B. afzelii* and *B. garinii* predominate, species-level attribution of cardiac disease is rarely available, and national Lyme borreliosis surveillance systems differ substantially in definitions, reporting routes, and captured manifestations. Available data suggest that Lyme carditis is rare among medically attended Lyme borreliosis cases but may be clinically relevant among patients with unexplained high-grade or fluctuating atrioventricular block in endemic regions. Diagnosis is complicated by absent tick-bite history or erythema migrans, early seronegativity, background seropositivity, older age at presentation, myocarditis or acute coronary syndrome-like phenotypes, and limited access to confirmatory tissue-based methods. We propose that European surveillance should incorporate cardiology-linked sentinel pathways, particularly before permanent pacemaker implantation, using standardized suspected, probable, and confirmed case categories supported by paired serology when feasible. Multicenter collaboration between cardiology, infectious diseases, microbiology, and public-health networks is needed to define incidence, optimize diagnostic algorithms, and clarify the contribution of European *Borrelia* species to cardiac involvement.

## 1 Introduction

Lyme borreliosis (LB) is the most prevalent tick-transmitted bacterial infection in temperate areas of Europe, North America, and Asia and is caused by members of the *Borrelia burgdorferi sensu lato* complex transmitted by infected *Ixodes* ticks (Stark et al., 2023). Untreated infection may disseminate to the nervous system, joints, and heart (Nagarajan et al., 2023).

Lyme carditis (LC) is among the less frequent extracutaneous LB manifestations, but its clinical importance is disproportionate to its frequency. The key reason is reversibility: high-grade atrioventricular block, sometimes progressing rapidly, may resolve after antibiotic therapy, whereas missed diagnosis can lead to unnecessary permanent pacemaker implantation or, rarely, fatal arrhythmia (Scheffold et al., 2015; Radesich et al., 2022; Halsby et al., 2026).

For Europe, LC should be framed as both a diagnostic and surveillance problem. Classical reviews report cardiac involvement in a minority of LB patients, but modern European prospective data suggest very low observed proportions among medically attended LB cases, while cardiology-based screening studies indicate that probable LC cases may be missed among selected pacemaker candidates in endemic regions (Kaczmarek et al., 2022; Gazibara et al., 2026; Halsby et al., 2026).

## 2 Lyme borreliosis in Europe, vectors, and human-pathogenic *Borrelia*

In most of Europe, *Ixodes ricinus* is the principal vector, while *Ixodes persulcatus* is relevant in parts of north-eastern Europe and Euroasia. Reservoir cycles involve *Ixodes* ticks and vertebrate hosts, especially small mammals and ground-feeding birds, whereas larger ungulates such as deer maintain tick populations without being competent *Borrelia* reservoirs (Nagarajan et al., 2023; Stark et al., 2023).

From a microbiology perspective, European LC is usually a species-unspecified manifestation of *Borrelia burgdorferi sensu lato* rather than a species-level diagnosis. Europe harbors a broader spectrum of human-pathogenic genospecies than North America, most consistently *B. afzelii*, *B. garinii*, *B. burgdorferi* sensu stricto, *B. ba*var*iensis*, and *B. spielmanii*, with regional variation in distribution, tissue tropism, and antigenic composition (Stanek et al., 2011, 2012; Kodym et al., 2018). Yet routine cardiac cases are rarely microbiologically typed because diagnosis is usually indirect and serum-based, not based on culture or tissue detection. This distinction is important for review framing: species-level attribution is generally not justified in individual LC reports, and the more defensible wording is Lyme carditis associated with *B. burgdorferi* s.l. infection. It also matters diagnostically, because the antigenic diversity of European strains influences assay design and contributes to inter-assay variability, particularly when tests developed around different antigen sets or regional strain backgrounds are compared (Kodym et al., 2018; Lager et al., 2019).

## 3 Epidemiology of Lyme carditis in Europe

The LC epidemiology must be interpreted against the background of heterogeneous LB surveillance. A review of national surveillance systems from 2005 to 2020 found estimates for 25 European countries, with marked differences in surveillance type, case definitions, and diagnostic criteria. Across systems, approximately 128,888 LB cases were reported annually, but comparability across countries was limited (Burn et al., 2023).

A separate review of European LB surveillance found that 28 of 36 assessed countries had surveillance in place, but systems varied in public availability, case definition, reporting route, granularity, and whether clinical manifestations were captured (Nagarajan et al., 2023). Lyme neuroborreliosis has a European manifestation-specific surveillance precedent (Blanchard et al., 2022), while LC does not yet have comparable routine capture.

In the prospective BOLD study of medically attended suspected LB in endemic regions of six European countries, erythema migrans dominated the clinical spectrum, whereas LC was rare. More specifically, three LC cases were reported among 797 LB cases in the abstract, and the detailed manifestation table reports 2–3 cases depending on classification/counting column (Halsby et al., 2026). This should not be interpreted as the full European LC incidence, because the study was not designed as a cardiology surveillance system for patients presenting primarily with conduction disease.

Cardiology-based studies provide a different denominator. In a Polish cohort of consecutive pacemaker candidates with symptomatic atrioventricular block, LC was considered the initial diagnosis in 16 of 130 patients (12%) after excluding obvious non-infectious causes (Kaczmarek et al., 2022). In northern Serbia, a prospective pacemaker-candidate study found serological patterns compatible with probable LC in 8 of 74 patients who completed follow-up (10.8%) (Gazibara et al., 2026).

Therefore, a useful epidemiological framework should distinguish three denominators: (i) all LB cases, where carditis appears rare; (ii) patients with unexplained high-grade or fluctuating atrioventricular block, where LC can be clinically meaningful in endemic areas; and (iii) surveillance systems, which currently do not capture LC consistently enough to estimate incidence.

## 4 Diagnostic challenges of Lyme carditis in Europe and possible manifestations

Lyme carditis is diagnostically difficult because it sits between infectious disease, electrophysiology, myocarditis imaging, emergency medicine, and public health surveillance. Patients may present with syncope, presyncope, dyspnea, chest pain, palpitations, bradycardia, atrial arrhythmias, myocarditis, or acute coronary syndrome-like features rather than with a remembered tick bite or typical erythema migrans (Scheffold et al., 2015).

The cardinal manifestation of LC is atrioventricular conduction disease. Reviews report that most LC patients develop conduction abnormalities, and a PR/PQ interval longer than 300 ms should trigger continuous rhythm monitoring because of the risk of high-grade block (Scheffold et al., 2015; Radesich et al., 2022). AV block may fluctuate rapidly, with progression from first-degree to complete block over hours or days.

Several pitfalls are especially relevant in Europe. First, early serology may be negative, whereas later seropositivity can represent prior exposure in endemic settings (Dessau et al., 2018). Second, erythema migrans and tick-bite history may be absent, as it is observed in 55–93.75% of European LC cases (Moerdijk et al., 2019; Kaczmarek et al., 2022). Third, older adults with atrioventricular block are often assumed to have degenerative conduction disease, although European pacemaker-candidate studies show that LC should not be restricted to the very young patients (Kaczmarek et al., 2022; Gazibara et al., 2026). Fourth, myocarditis or myopericarditis can mimic acute coronary syndrome when troponin is elevated and ECG findings are non-specific. Finally, the heterogeneous distribution of *Borrelia* species in Europe, particularly *B. afzelii* and *B. garinii*, may contribute to clinical variability compared with North American LC. Also, there is no universally accepted European case definition for LC, resulting in probable underrecognition and inconsistent reporting across countries. In addition, cardiac magnetic resonance imaging and endomyocardial biopsy with immuno-histological evidence of typical lymphocytic myocarditis and detection of spirochaete by PCR, may support diagnosis in selected cases, but their findings are non-specific and rarely available in routine clinical practice (Kaczmarek et al., 2022; Schroeter et al., 2022).

Accordingly, the microbiological diagnosis of LC in Europe is supportive and contextual rather than standalone. Two-tier serology remains the main laboratory approach for extracutaneous LB in Europe, typically with a sensitive screening immunoassay followed by confirmatory immunoblot, but the result must be interpreted against syndrome, timing, and pre-test probability (Scheffold et al., 2015; Dessau et al., 2018; Radesich et al., 2022). Early seronegativity remains possible during the first weeks of infection, whereas background seropositivity in endemic populations means that a single positive serum sample may reflect previous exposure rather than active cardiac infection (Hillerdal and Henningsson, 2021; Hoeve-Bakker et al., 2024). This is particularly relevant in Europe, where population seropositivity increases with age and exposure, and where isolated IgM reactivity can be misleading if used outside an appropriately early clinical context (Hillerdal and Henningsson, 2021; Hoeve-Bakker et al., 2024). When initial testing is negative but suspicion remains high, repeat serology or paired samples demonstrating seroconversion or rising/stable IgG can strengthen causality, as illustrated in recent European conduction-disease cohorts (Dessau et al., 2018; Gazibara et al., 2026). PCR or histopathology can provide stronger microbiological support when endomyocardial biopsy, surgical tissue, or autopsy material is available, but these are specialist, non-screening investigations and a negative PCR does not exclude LC (Dessau et al., 2018; Radesich et al., 2022).

The Suspicious Index in Lyme Carditis (SILC) score, which was introduced by a Canadian group of cardiologists, is useful as a structured bedside tool, but European modification may be needed. The Polish pacemaker-candidate study evaluated modified SILC variants that incorporated age below 75 years, countryside/endemic exposure, and fluctuating AV block, highlighting the need to adapt prediction tools to European demographics and exposure patterns (Kaczmarek et al., 2022).

European pacing guidance is directly relevant. The 2021 ESC pacing guidelines state that pacing is not recommended in AV block due to transient causes that can be corrected and prevented, and recommend temporary transvenous pacing in haemodynamically compromising bradyarrhythmia or when pacing indications are expected to be reversible, such as myocarditis (Glikson et al., 2021). In suspected LC, this supports telemetry, prompt antibiotics, temporary pacing when needed, and reassessment before permanent pacemaker implantation.

### 4.1 Synthesis of manifestations represented in the case literature

The European case literature is dominated by AV conduction disease, especially fluctuating high-grade or complete AV block (Supplementary Table 1). However, the phenotype is broader: sinus node dysfunction, sinoatrial exit block, atrial fibrillation/flutter, ventricular tachyarrhythmia including torsades/cardiac arrest, acute coronary syndrome mimics, myocarditis, cardiogenic shock, and Cardiac Magnetic Resonance (CMR) -documented myocardial inflammation are all represented (Moerdijk et al., 2019; Javed et al., 2024; Kaczynski et al., 2024)

## 5 Proposal for improvement of surveillance of Lyme carditis in Europe

A realistic European surveillance strategy should start with sentinel, cardiology-linked surveillance in endemic and emerging-risk regions, harmonized with existing LB and Lyme neuroborreliosis surveillance. The aim should be to detect clinically consequential, potentially reversible cardiac disease rather than to overburden general Lyme reporting systems. Categories presented in Table 1 should explicitly separate active/probable LC from past *Borrelia* exposure, because seropositivity alone is insufficient in endemic European populations.

**Table 1:** Proposal of a European working case definition for Lyme carditis surveillance.

Surveillance category	Proposed definition
Suspected Lyme carditis	New compatible cardiac manifestation - especially new second-degree, high-grade, or third-degree AV block; rapidly fluctuating PR interval or AV block; unexplained myocarditis/myopericarditis; ventricular arrhythmia; atrial arrhythmia with conduction disease; or ACS mimic - plus residence, travel, or outdoor exposure in a Lyme-endemic area.
Probable Lyme carditis	Suspected case plus positive two-tier serology, recent erythema migrans, compatible systemic Lyme symptoms, or paired serology showing seroconversion or stable/rising titers, with no stronger alternative diagnosis.
Confirmed Lyme carditis	Compatible cardiac manifestation plus laboratory evidence supporting active Lyme borreliosis, such as seroconversion, PCR/histopathological evidence from cardiac tissue in exceptional cases, or strong clinical-serological evidence with documented resolution after antibiotics and exclusion of alternative causes.

Participating centers should screen a standardized subset of patients before permanent pacemaker implantation: new high-grade AV block, third-degree AV block, unexplained fluctuating AV conduction, AV block with constitutional symptoms, AV block with myocarditis biomarkers, or AV block without clear degenerative, ischemic, drug-related, electrolyte, or procedural cause. This approach is supported by the Polish and Serbian pacemaker-candidate studies, both of which identified clinically meaningful proportions of possible/probable LC in selected pacemaker pathways (Kaczmarek et al., 2022; Gazibara et al., 2026).

Surveillance should specify the diagnostic algorithm used, the antigen panel, and whether the case is based on single or paired serology. The Serbian study is instructive because it used baseline and four-week follow-up sera and defined probable LC using IgG seroconversion or stable/rising titres, while distinguishing persistent seronegativity or declining titres (Gazibara et al., 2026). Implementation of relatively cheap ELISA in diagnostic work-up for every patient with cardiac manifestation would help in real-time detection of LC cases, as well as in providing a baseline for future surveillance.

European LB surveillance systems already vary substantially, but several countries report dashboards or reports with granular data. A LC module could be added to participating systems and linked to electrophysiology registries, microbiology laboratories, infectious disease surveillance networks, and myocarditis/CMR workflows (Nagarajan et al., 2023).

## 6 Discussion

Lyme carditis in Europe remains clinically important but epidemiologically under-defined. The first major controversy concerns burden: LC appears rare in unselected LB cohorts, yet cardiology-based studies in patients with unexplained atrioventricular block or pacemaker indications suggest that probable cases may be more frequent than traditionally assumed. This discrepancy likely reflects heterogeneous national surveillance systems, the absence of a harmonized LC case definition, and the use of very different clinical denominators across infectious disease and electrophysiology settings (Kaczmarek et al., 2022; Burn et al., 2023; Nagarajan et al., 2023; Gazibara et al., 2026).

A second unresolved issue is diagnostic attribution. In endemic European settings, positive *Borrelia* serology may indicate previous exposure rather than active cardiac infection, whereas early infection can still be seronegative. In addition, many patients with LC do not recall a tick bite or erythema migrans, and currently used clinical tools may require European validation, particularly in older patients presenting through pacemaker pathways rather than through classical early-disseminated LB pathways. These factors help explain why diagnostic uncertainty persists even though the cardiac phenotype, especially rapidly fluctuating high-grade atrioventricular block, is often highly suggestive once recognized (Kaczmarek et al., 2022; Radesich et al., 2022; Hoeve-Bakker et al., 2024; Gazibara et al., 2026).

Future work should focus on prospective multicenter studies with harmonized suspected/probable/confirmed case definitions, paired serology where feasible, systematic rhythm phenotyping, and clearer guidance on temporary pacing, permanent pacemaker avoidance, and the role of cardiac magnetic resonance in atypical or myocarditic presentations. A cardiology-linked surveillance approach would be particularly valuable in endemic regions, where delayed recognition may lead to avoidable permanent device implantation (Radesich et al., 2022; Nagarajan et al., 2023; Kaczynski et al., 2024; Gazibara et al., 2026).

In conclusion, LC in Europe is uncommon but high-impact: it is usually reversible, occasionally life-threatening, and still insufficiently captured by current surveillance frameworks.
