# Supplementary material for: Lyme carditis in Europe
Source: Front Microbiol. 2026 Jul 30;17:1905414. doi: 10.3389/fmicb.2026.1905414 (PMC13469242; doi:10.3389/fmicb.2026.1905414)
Supplement: Supplementary file 1 [file Table_1.docx]

The review was developed as a targeted narrative review informed by the references supplied by the authors, targeted PubMed/MEDLINE searches, hand-searching of reference lists of key reviews and case reports, and focused checks of official public-health and journal websites for European surveillance data and recent publications. Searches and checks covered material available up to 10 June 2026, with practical emphasis on publications from 2000 onward for European case reports, surveillance studies, and clinically relevant reviews or guidelines. Search terms combined Lyme carditis, Lyme borreliosis, Borrelia burgdorferi sensu lato, Europe, atrioventricular block, myocarditis, arrhythmia, conduction disease, pacemaker, and selected country names.

Priority was given to human studies relevant to Europe, including case reports and small case series, observational studies, surveillance analyses, guideline or consensus documents, and selected reviews used for clinical or epidemiologic context. Non-European publications were used sparingly when they clarified diagnosis, pathophysiology, imaging, or management issues insufficiently covered by European data. Because the manuscript was designed as a narrative review, no pre-registered protocol, duplicate screening, formal risk-of-bias assessment, or PRISMA flow diagram was used. The resulting synthesis therefore remains vulnerable to publication bias, language bias, selective reporting, duplicate publication risk in case-based literature, and heterogeneity in surveillance definitions and ascertainment across European countries.

# Supplementary Table 1. Published European case reports and small case series of Lyme carditis. Country refers to the country of report or clinical management unless exposure was specified elsewhere. Etiology is usually reported as Lyme borreliosis / B. burgdorferi sensu lato by serology; species-level identification is uncommon.

| **Manifestation** | **Etiology / diagnostic evidence** | **No. cases** | **Country** | **Reference / source note** |
| --- | --- | --- | --- | --- |
| Total atrioventricular block requiring pacemaker implantation | Lyme borreliosis; details limited in indexed source | 1 | Netherlands | (Kuiper et al., 1988) |
| Lyme carditis with conduction involvement | Lyme borreliosis / *B. burgdorferi* sensu lato, reported as Lyme carditis | 1 | Netherlands | (Blaauw et al., 1989) |
| Transient complete AV block with electrophysiologic observations | Lyme borreliosis / *B. burgdorferi* s.l. | 1 | Netherlands | (van der Linde et al., 1989) |
| Complete heart block requiring temporary pacing | Lyme borreliosis / *B. burgdorferi* s.l. | 1 | Hungary | (Lórincz et al., 1989) |
| Persistent AV block | Lyme borreliosis | 1 | Germany | (Mayer et al., 1990) |
| Fatal Lyme carditis with AV nodal pathology | Fatal Lyme carditis; post-mortem/histopathologic context | 1 | United Kingdom | (Cary et al., 1990) |
| Irreversible complete heart block | Lyme borreliosis / *B. burgdorferi* s.l. | 1 | Spain | (Artigao et al., 1991) |
| Transitory AV block | Cardiac manifestation of Lyme borreliosis | 1 | Germany | (Siebenlist et al., 1991) |
| Transient complete AV block | *Borrelia* myocarditis | 1 | Germany | (Kruse et al., 1992) |
| Complete heart block with syncope | Lyme borreliosis carditis | 3 | Serbia / former Yugoslavia | (Vasiljević et al., 1996) |
| Temporary decrease in heart rate during antibiotic treatment | Lyme carditis | 1 | Netherlands | (Dam et al., 1996) |
| Sick sinus syndrome / sinus-node involvement | *B. burgdorferi* infection reported as cause | 1 | Czech Republic | (Bartůnĕk et al., 1996) |
| Complete transient AV block | Lyme borreliosis | 1 | Spain | (Batalla Celorio et al., 1999) |
| Acute coronary syndrome / anterior myocardial infarction mimic | *Borrelia* myocarditis / Lyme carditis | 1 | Germany | (Dernedde et al., 2002) |
| AV block | Lyme carditis | 1 | Turkey | (Akyol et al., 2003) |
| Symptomatic sinus node dysfunction | Lyme carditis | 1 | Germany | (Franck and Wollschläger, 2003) |
| AV block | Lyme carditis | 1 | Switzerland | (Karadag et al., 2004) |
| AV block | Lyme carditis | 1 | Switzerland | (Pasquier et al., 2006) |
| Complete AV dissociation / third-degree AV block requiring temporary transvenous pacing | Lyme borreliosis; resolved after doxycycline; exposure history complex | 1 | Greece | (Xanthos et al., 2006) |
| AV block / Lyme carditis phenotype | Lyme carditis | 1 | Belgium | (Beuselinck and Willems, 2007) |
| AV block / conduction disease | Lyme carditis | 2 | Poland | (Elikowski et al., 2007) |
| Transient total AV block with myocardial injury; persistent local late gadolinium enhancement on CMR | Isolated Lyme myocarditis / Lyme carditis | 1 | Norway | (Munk et al., 2007) |
| AV block | Lyme carditis | 1 | Netherlands | (Verbunt and Visser, 2007) |
| Lyme carditis with diagnostic delay emphasised | Lyme carditis | 1 | Poland | (Legatowicz-Koprowska et al., 2007) |
| Acute coronary syndrome mimic | Lyme carditis / probable myopericarditis | 1 | Poland | (Rostoff et al., 2008) |
| Reversible complete AV block during reinfection; IgM-negative presentation | Lyme carditis / reinfection | 1 | Germany | (Guenther et al., 2009) |
| Complete AV block; reversible | Lyme carditis | 1 | Scotland / United Kingdom | (Bhattacharya et al., 2010) |
| Complete AV block | Lyme carditis | 1 | Germany | (Semmler et al., 2010) |
| Complete AV block with presyncope; supra-Hisian block; recovery without permanent pacemaker | Serologically verified Lyme borreliosis; older infection suggested | 1 | Hungary | (Wagner et al., 2010) |
| High-grade / complete AV block requiring temporary pacing | Lyme carditis after tick bite; resolved with antibiotics | 1 | Italy | (Bacino et al., 2011) |
| AV block as first presentation of disseminated Lyme disease | Disseminated Lyme borreliosis / *B. burgdorferi* s.l. | 1 | Serbia | (Panić et al., 2011) |
| Atrial fibrillation, complete AV block, escape rhythm with bundle-branch block morphologies | Lyme carditis | 1 | Switzerland | (Wenger et al., 2012) |
| AV block / Lyme carditis phenotype | Lyme carditis | 1 | Poland | (Dobaj et al., 2012) |
| Complete AV block as first symptom; antibiotics used instead of pacemaker implantation | Lyme carditis / Lyme borreliosis | 1 | Poland | (Kaczmarek et al., 2013) |
| Unclassified cardiomyopathy vs Lyme carditis; three-year follow-up | Possible Lyme carditis / cardiomyopathy differential | 1 | Poland | (Konopka et al., 2013) |
| Reversible total AV block; diagnosis aided by photograph of erythema migrans | *B. burgdorferi* infection / Lyme carditis | 1 | Germany | (Brunner et al., 2014) |
| Third-degree AV block complicated by torsades de pointes ventricular tachycardia and cardiac arrest | Lyme carditis | 1 | Denmark | (Jensen et al., 2014) |
| AV block / Lyme carditis phenotype | Lyme carditis | 1 | Croatia | (Kurtić et al., 2014) |
| Lyme carditis / AV-block phenotype | Lyme disease with cardiac involvement | 1 | Switzerland | (Arroja et al., 2015) |
| AV block / Lyme carditis phenotype | Lyme carditis | 1 | Netherlands | (van Hattem et al., 2015) |
| AV-block Lyme carditis cases identified in national / GP-linked data | Lyme carditis | 4 | Netherlands | (Hofhuis et al., 2015) |
| Pediatric AV block / Lyme carditis phenotype | Lyme carditis | 1 | Belgium | (Muschart and Blommaert, 2015) |
| Perioperative cardiogenic shock suggesting acute coronary syndrome | Lyme carditis / myocarditis | 1 | Belgium | (Clinckaert et al., 2016) |
| Reversible third-degree AV block | Lyme carditis | 1 | Netherlands | (Timmer et al., 2016) |
| Fulminant myocarditis with atrial flutter, severe biventricular failure, cardiogenic shock, multiorgan failure; mechanical circulatory support | Disseminated *Borrelia* infection confirmed serologically | 1 | Croatia | (Župan et al., 2017) |
| Second-degree AV block | Lyme borreliosis / Lyme carditis | 1 | Netherlands | (van der Zande et al., 2020) |
| Dyspnoea/palpitations; first-degree AV block and frequent atrial extrasystoles progressing to intermittent complete AV block within 48 h | Lyme carditis confirmed by serology; prior untreated tick bite reported | 1 | Germany | (Matthiae and Kriatselis, 2021) |
| Reversible high-grade AV block with septal myocardial oedema | Lyme carditis | 1 | Germany | (Prochnau et al., 2022) |
| Sinoatrial exit block with asystole >12 s and syncope; resolved after ceftriaxone | Lyme carditis with positive IgM/IgG immunoblot | 1 | Germany | (Büscher et al., 2022) |
| Two diagnostic-delay cases: Mobitz I progressing to third-degree AV block requiring temporary pacing; myopericarditis / CMR-confirmed myocarditis phenotype | Lyme carditis / Lyme myocarditis in young males from endemic western Norway | 2 | Norway | (Sørås et al., 2025) |

**Abbreviations:** AV = atrioventricular; CMR = cardiac magnetic resonance; LGE = late gadolinium enhancement; s.l. = sensu lato. The table emphasizes clinical phenotype and publication traceability rather than formal incidence.

**References**

Akyol, A., Burgun, A., Eksik, A., Çakmak, N., Oğuz, E., Erdinler, İ., et al. (2003). Transient Complete AV Block due to Lyme Carditis. *Archives of the Turkish Society of Cardiology* 31, 466–470.

Arroja, J. D., Fassa, A.-A., and Zimmermann, M. (2015). Lyme carditis. *Rev Med Suisse* 11, 563–566, 568.

Artigao, R., Torres, G., Guerrero, A., Jiménez-Mena, M., and Bayas Paredes, M. (1991). Irreversible complete heart block in Lyme disease. *Am J Med* 90, 531–533.

Bacino, L., Gazzarata, M., Siri, G., Cordone, S., and Bellotti, P. (2011). Blocco atrioventricolare completo come prima presentazione clinica di puntura di zecca (malattia di Lyme). *Giornale Italiano di Cardiologia* 12, 214–216.

Bartůnĕk, P., Nĕmec, J., Mrázek, V., Gorican, K., and Zapletalová, J. (1996). Borrelia burgdorferi as a cause of sick sinus syndrome?. *Cas Lek Cesk* 135, 729–731.

Batalla Celorio, A., Martínez González, J., González Camino, F., Suárez Suárez, E., Hevia Nava, S., and Barriales Álvarez, V. (1999). Bloqueo auriculoventricular completo transitorio en la enfermedad de Lyme. *Revista Española de Cardiología* 52, 529–531. doi: 10.1016/S0300-8932(99)74963-8

Beuselinck, B., and Willems, R. (2007). Think outside the heart! Case report of Lyme disease. *Acta Cardiologica* 62, 519–522. doi: 10.2143/AC.62.5.2023418

Bhattacharya, I., Dweck, M., and Francis, M. (2010). Lyme carditis: a reversible cause of complete atrioventricular block. *Journal of the Royal College of Physicians of Edinburgh* 40, 121–122. doi: 10.4997/JRCPE.2010.207

Blaauw, A. A., van der Linden, Sj., and Kuiper, H. (1989). Lyme Carditis in the Netherlands. *Ann Intern Med* 111, 261–262. doi: 10.7326/0003-4819-111-3-261_2

Brunner, F. J., Blankenberg, S., and Sydow, K. (2014). Digital camera revealed infection with Borrelia burgdorferi as a cause of reversible total AV block in a 42 year old man. *International Journal of Cardiology* 177, e165–e166. doi: 10.1016/j.ijcard.2014.08.139

Büscher, A., Doldi, F., Eckardt, L., and Müller, P. (2022). Lyme carditis manifesting with sinoatrial exit block: a case report. *Eur Heart J - Case Rep* 6, ytac022. doi: 10.1093/ehjcr/ytac022

Cary, N. R., Fox, B., Wright, D. J., Cutler, S. J., Shapiro, L. M., and Grace, A. A. (1990). Fatal Lyme carditis and endodermal heterotopia of the atrioventricular node. *Postgrad Med J* 66, 134–136. doi: 10.1136/pgmj.66.772.134

Clinckaert, C., Bidgoli, S., Verbeet, T., Attou, R., Gottignies, P., Massaut, J., et al. (2016). Peroperative cardiogenic shock suggesting acute coronary syndrome as initial manifestation of Lyme carditis. *J Clin Anesth* 35, 430–433. doi: 10.1016/j.jclinane.2016.08.005

Dam, E. P., Jonker, D. R., and Hoorntje, J. C. (1996). Temporary decrease in heart rate in Lyme carditis during treatment with antibiotics. *Heart* 76, 289–290. doi: 10.1136/hrt.76.3.289

Dernedde, S., Piper, C., Kühl, U., Kandolf, R., Mellwig, K. P., Schmidt, H. K., et al. (2002). Borrelien-Myokarditis als seltene Differentialdiagnose des akuten Vorderwandinfarktes. *Z Kardiol* 91, 1053–1060. doi: 10.1007/s00392-002-0873-4

Dobaj, Ł., Bartkowiak, R., Bąkowski, D., and Wożakowska-Kapłon, B. (2012). Całkowity blok przedsionkowo-komorowy w przebiegu boreliozy z Lyme. *Choroby Serca i Naczyń* 9, 44–49.

Elikowski, W., Małek, M., Flieger, J., Baszko, A., Fagiewicz, A., and Wróblewski, D. (2007). Complete heart block and Lyme disease: report of two different cases and literature review. *Kardiol Pol* 65, 565–570.

Franck, H., and Wollschläger, H. (2003). SymptomatischeSinusknotendysfunktion bei Lyme-Karditis. *Z Kardiol* 92, 1029–1032. doi: 10.1007/s00392-003-0995-3

Guenther, F., Bode, C., and Faber, T. (2009). Reversibler totaler AV-Block durch Reinfektion mit Borrelien bei negativen IgM-Titern. *Dtsch Med Wochenschr* 134, 23–26. doi: 10.1055/s-0028-1105885

Hofhuis, A., Arend, S. M., Davids, C. J., Tukkie, R., and van Pelt, W. (2015). General practitioner reported incidence of Lyme carditis in the Netherlands. *Neth Heart J* 23, 533–538. doi: 10.1007/s12471-015-0744-z

Jensen, T. B., Dalsgaard, D., and Johansen, J. B. (2014). Cardiac arrest due to torsades de pointes ventricular tachycardia in a patient with Lyme carditis. *Ugeskr Laeger* 176, V03140168.

Kaczmarek, K., Deroń, Z., Janiszewski, T., Supeł, K., Ptaszyński, P., Ruta, J., et al. (2013). Complete atrio-ventricular block as a first symptom of borreliosis: antibiotic treatment instead of pacemaker implantation. *Kardiol Pol* 71, 205–207. doi: 10.5603/KP.2013.0020

Karadag, B., Spieker, L. E., Schwitter, J., Ruschitzka, F., Lüscher, T. F., Noll, G., et al. (2004). Lyme Carditis: Restitutio Ad Integrum Documented by Cardiac Magnetic Resonance Imaging. *Cardiology in Review* 12, 185. doi: 10.1097/01.crd.0000123841.02777.5d

Konopka, M., Kuch, M., Braksator, W., Walczak, E., Jakuciński, M., Lipowski, D., et al. (2013). Unclassified cardiomyopathy or Lyme carditis? A three year follow-up. *Kardiol Pol* 71, 283–285. doi: 10.5603/KP.2013.0043

Kruse, K. M., Gust, R., and Grosse-Heitmeyer, W. (1992). Transient complete AV block as a sequela of Borrelia myocarditis. *Dtsch Med Wochenschr* 117, 167–171. doi: 10.1055/s-2008-1062294

Kuiper, H., de Jongh, B. M., and Senden, P. J. (1988). Pacemaker implantation for complete atrioventricular block due to Lyme borreliosis. *Ned Tijdschr Geneeskd* 132, 2109–2111.

Kurtić, E., Premužić Meštrović, I., Zeljko, H. M., Kranjčević, S., Kranjčević, D., and Jerkić, H. (2014). Pristup bolesniku s poremećajem provođenja uzrokovan Lyme boreliozom. *Cardiologia Croatica* 9, 40–43. doi: 10.15836/ccar.2014.40

Legatowicz-Koprowska, M., Gziut, A. I., Jezierski, J., Ząbek, J., Lipowski, D., Walczak, E., et al. (2007). Case report Lyme carditis &#8211; a bitter lesson or a delayed diagnostic success &#8211; a case report. *Polish Heart Journal (Kardiologia Polska)* 65, 1228–1230. doi: 10.33963/v.kp.81104

Lórincz, I., Lakos, A., Kovács, P., Várvölgyi, C., Polgár, P., and Wórum, F. (1989). Temporary pacing in complete heart block due to Lyme disease: a case report. *Pacing Clin Electrophysiol* 12, 1433–1436. doi: 10.1111/j.1540-8159.1989.tb05059.x

Matthiae, D., and Kriatselis, C. (2021). [Dyspnea and palpitations in a 39-year-old male patient]. *Internist (Berl)* 62, 871–875. doi: 10.1007/s00108-021-01047-4

Mayer, W., Kleber, F. X., Wilske, B., Preac-Mursic, V., Maciejewski, W., Sigl, H., et al. (1990). Persistent atrioventricular block in Lyme borreliosis. *Klin Wochenschr* 68, 431–435. doi: 10.1007/BF01648587

Munk, P. S., Ørn, S., and Larsen, A. I. (2007). Lyme carditis: persistent local delayed enhancement by cardiac magnetic resonance imaging. *Int J Cardiol* 115, e108-110. doi: 10.1016/j.ijcard.2006.05.068

Muschart, X., and Blommaert, D. (2015). Seeks, finds, threats: Lyme disease! *Am J Emerg Med* 33, 307.e5–6. doi: 10.1016/j.ajem.2014.07.019

Panić, G., Stanulović, V., and Popov, T. (2011). Atrio-ventricular block as the first presentation of disseminated Lyme disease. *International Journal of Cardiology* 150, e104–e106. doi: 10.1016/j.ijcard.2010.02.061

Pasquier, M., Péter, O., Frochaux, V., Imsand, C., Vogt, P., and Girod, G. (2006). Bloc atrioventriculaire complet sur borréliose de Lyme: aspects etectrocardiographiques, diagnostiques et microbiologiques [Atrioventricular heart block in Lyme disease]. 2, 415–417.

Prochnau, D., Kühnemund, J., and Heyne, J. P. (2022). Reversible high-grade atrioventricular block with septal myocardial edema in Lyme carditis. *Herzschrittmacherther Elektrophysiol* 33, 327–329. doi: 10.1007/s00399-022-00879-3

Rostoff, P., Konduracka, E., El Massri, N., Gackowski, A., Kruszec, P., Zmudka, K., et al. (2008). Lyme carditis presenting as acute coronary syndrome: a case report. *Kardiol Pol* 66, 420–425.

Semmler, D., Blank, R., and Rupprecht, H.-J. (2010). Complete AV block in Lyme carditis: an important differential diagnosis. *Clin Res Cardiol* 99, 519–526. doi: 10.1007/s00392-010-0152-8

Siebenlist, D., Hahn, M., and Gattenlöhner, W. (1991). Transitory AV block as a cardiac manifestation of Lyme disease. *Dtsch Med Wochenschr* 116, 1549–1552. doi: 10.1055/s-2008-1063787

Sørås, J. A., Svingen, G. F. T., Larsen, T. H., Keilegavlen, H., Kristiansen, T., and Wendelbo, Ø. (2025). Diagnostic delay in patients diagnosed with Lyme carditis presenting with cardiac symptoms. *J Cardiol Cases* 32, 19–22. doi: 10.1016/j.jccase.2025.03.011

Timmer, S. A. J., Boswijk, D. J., Kimman, G. P., and Germans, T. (2016). A case of reversible third-degree AV block due to Lyme carditis. *Journal of Electrocardiology* 49, 519–521. doi: 10.1016/j.jelectrocard.2016.05.006

van der Linde, M. R., Crijns, H. J., and Lie, K. I. (1989). Transient complete AV block in Lyme disease. Electrophysiologic observations. *Chest* 96, 219–221. doi: 10.1378/chest.96.1.219

van der Zande, J. M. J., Baarsma, M. E., Grundeken, M. J., Verhaar, N., Kok, W. E. M., and Hovius, J. W. (2020). [Second-degree atrioventricular block caused by Lyme disease]. *Ned Tijdschr Geneeskd* 164, D4214.

van Hattem, J. M., Keijer, J. T., and Nijveldt, R. (2015). A man with cardiac Lyme borreliosis. *CMAJ* 187, 1071–1073. doi: 10.1503/cmaj.141468

Vasiljević, Z., Dmitrović, R., Naumović, Z., Ostojić, M., Radosavljević, M., Karadzić, A., et al. (1996). Common form of Lyme borreliosis carditis--complete heart block with syncope: report on 3 cases. *Cardiology* 87, 76–78. doi: 10.1159/000177064

Verbunt, R. J. a. M., and Visser, R. F. (2007). [Total atrioventricular block following a tick bite]. *Ned Tijdschr Geneeskd* 151, 1941–1944.

Wagner, V., Zima, E., Gellér, L., and Merkely, B. (2010). Acute atrioventricular block in chronic Lyme disease. doi: 10.1556/oh.2010.28965

Wenger, N., Pellaton, C., Bruchez, P., and Schläpfer, J. (2012). Atrial fibrillation, complete atrioventricular block and escape rhythm with bundle-branch block morphologies: An exceptional presentation of Lyme carditis. *International Journal of Cardiology* 160, e12–e14. doi: 10.1016/j.ijcard.2012.01.004

Xanthos, T., Lelovas, P., Kantsos, H., Dontas, I., Perrea, D., and Kouskouni, E. (2006). Lyme carditis: complete atrioventricular dissociation with need for temporary pacing. *Hellenic J Cardiol* 47, 313–316.

Župan, Ž., Mijatović, D., Medved, I., Kraljić, S., Juranić, J., Barbalić, B., et al. (2017). Successful treatment of fulminant Lyme myocarditis with mechanical circulatory support in a young male adult: a case report. *Croat Med J* 58, 185–193. doi: 10.3325/cmj.2017.58.185
